# The E3 ubiquitin ligase SPRYD3-MYCBP2(PAM) regulates mitotic cell fate and ubiquitination of USP11 to control spindle assembly

**DOI:** 10.1016/j.jbc.2025.110785

**Published:** 2025-10-04

**Authors:** Alexandra Rita Turi da Fonte Dias, Ingrid Hoffmann

**Affiliations:** 1 Cell Cycle Control and Carcinogenesis, Heidelberg, Germany; 2 Faculty of Biosciences, Heidelberg University, Heidelberg, Germany

**Keywords:** deubiquitinase, E3 ligase, mitotic cell fate, MYCBP2, spindle assembly, SPRYD3, ubiquitination, USP11

## Abstract

MYCBP2 (PAM) is a large signaling hub that plays a key role in various processes, including neuronal connectivity and growth, cell division, and protein ubiquitination. Together with the substrate specificity factor FBXO45, MYCBP2 forms an E3 ligase complex that is involved in mitotic cell fate decision. During extended mitotic arrest caused by anti-microtubule drugs, cells may either experience cell death or escape mitosis through mitotic slippage. E3 ligase mediated ubiquitination is antagonized by deubiquitinating enzymes (DUBs). In this study, we show that despite their opposing activities, DUB-E3 ligase complexes can form and cooperate. We identify an E3 ligase complex consisting of MYCBP2 and a new substrate specificity factor, SPRYD3. Interestingly, SPRYD3-MYCBP2 promotes bipolar spindle formation by facilitating non-canonical ubiquitination on the DUB USP11 cysteine 318. We find that this process promotes bipolar spindle formation and mitotic slippage in presence of microtubule targeting drugs.

Ubiquitination is a post-translational modification that governs various aspects of eukaryotic biology (1). The initiation of ubiquitination occurs by transfer of ubiquitin (Ub) from an ubiquitin-activating enzyme (E1) to a ubiquitin-conjugating enzyme (E2). The E2–Ub complex is then recruited by an E3 ubiquitin ligase, which in case of RING-type E3 ligases mediates the direct transfer of ubiquitin from the E2 to a substrate. Ubiquitination is commonly regarded as a post-translational modification of lysine residues.

MYCBP2 (also known as PHR (PAM/Highwire/RPM-1)) plays important roles in synaptogenesis, axon termination (2, (3) and in autophagy by targeting ULK kinase (4). MYCBP2 is an atypical RING-type E3 ligase as it uses a novel ‘Ring-Cys-relay’ (RCR) catalytic mechanism and has no activity against lysine residues (5, 6). Instead, it stabilizes the axon survival factor NMNAT2 by targeting serine and threonine residues (6, 7). Both MYCBP2 and its *C. elegans* ortholog RPM-1 interact with the F-box protein FBXO45, an ubiquitination substrate selector (7, 8). FBXO45 contains a conserved F-box domain and a SPRY-domain (9), both of which play a role in recruiting substrates to the ubiquitin ligase complex (10, 11, 12).

Deubiquitinases (DUBs) and E3 ligases work together to coordinate ubiquitin signaling by ensuring selectivity for various substrates and/or ubiquitin signals (13, 14). Ubiquitin-specific protease 11 (USP11) belongs to the ubiquitin-specific protease (USP) family. USP11 augments pathological tau aggregation upon deubiquitination in female but not in male brain tissues from Alzheimer’s disease patients (15). USP11 also plays a tumor-promoting role in several cancer types (16, 17). For example, USP11 contributes to the progression of prostate cancer by upregulating c-Myc and androgen receptor activity (18).

Proper function of the mitotic spindle is required for successful cell division. Disruption of microtubule dynamics triggers the activation of the spindle assembly checkpoint (SAC) and leads to mitotic arrest. However, cells can bypass spindle assembly checkpoint-induced mitotic arrest through a process known as mitotic slippage (19). Upon mitotic arrest caused by anti-microtubule drugs, FBXO45-MYCBP2 functions in mitotic cell fate decision by ubiquitinating the tumor suppressor FBXW7, which leads to FBXW7 degradation and mitotic slippage (11). Given the high mechanistic diversity observed for E3 enzymes, it is of high interest to unravel new functions of MYCBP2.

Here we show that the atypical E3 ligase MYCBP2 binds to the SPRY-domain containing protein SPRYD3 to form a new E3 ubiquitin ligase complex. We identified the DUB USP11 as a down-stream target of SPRYD3-MYCBP2. Interestingly, SPRYD3-MYCBP2 activity results in ubiquitination on a cysteine residue, C318, thus promoting proper bipolar spindle formation and mitotic slippage in cells treated with microtubule poisons.

## Results

### SPRYD3 is a new MYCBP2(PAM)-interacting protein

**Figure 1:**
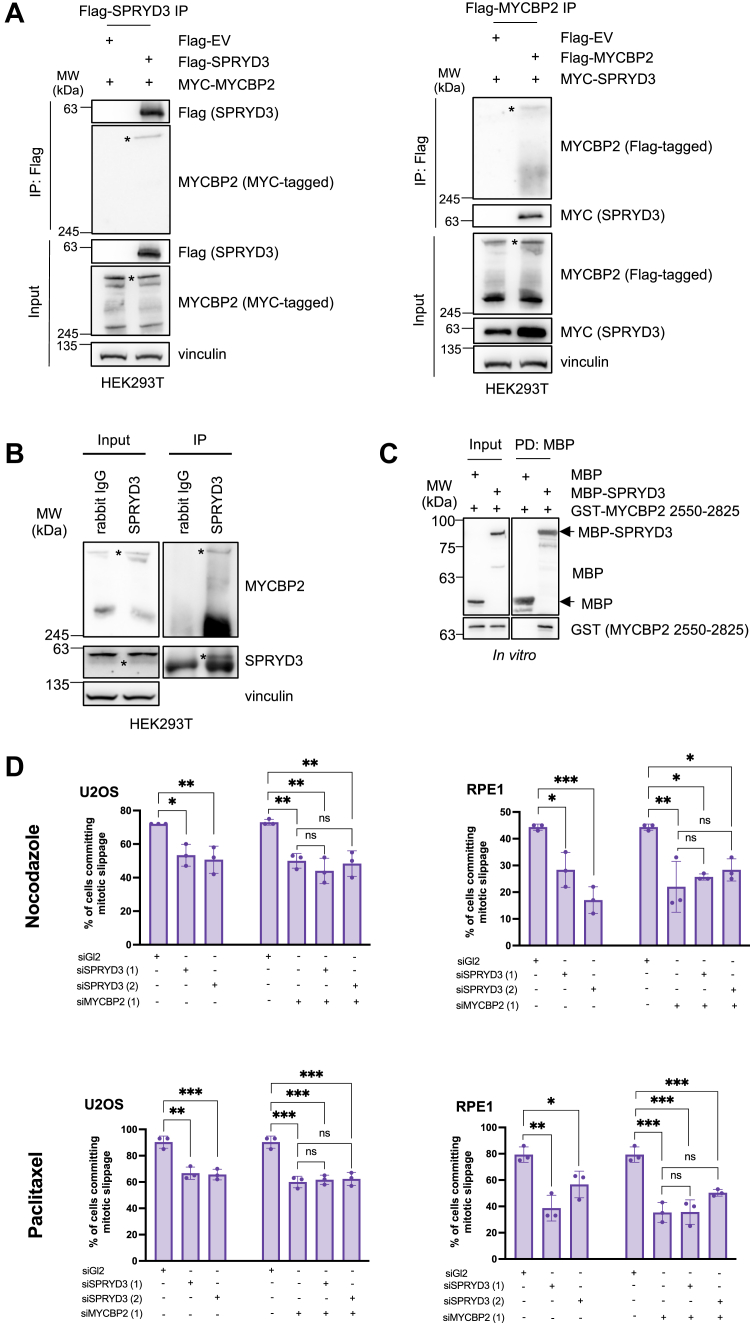
**SPRYD3 is a new interactor of the E3 ligase MYCBP2.***A*, Flag-SPRYD3 and MYC-MYCBP2 or Flag-MYCBP2 and MYC-SPRYD3 were co-transfected into HEK293T WT cells as indicated and overexpressed for 24 h (*left*) or 48 h (*right*). Cells were harvested for Flag-immunoprecipitation (Flag-IP). Western blot analyses demonstrate reciprocal interaction of ectopically expressed MYCBP2 and SPRYD3. ∗ indicates MYCBP2. *B*, endogenous SPRYD3 was immunoprecipitated by incubating HEK293T WT cell lysates with anti-IgG (*control*) or anti-SPRYD3 antibodies and Protein A sepharose. Western blot confirms co-immunoprecipitation of endogenous MYCBP2. ∗ indicates MYCBP2 or SPRYD3 respectively. *C*, recombinant MBP or MBP-SPRYD3 was combined with GST-MYCBP2 2550 to 2825 to perform an MBP pull-down. Western blot analysis confirms direct interaction between recombinant SPRYD3 and MYCBP2 2550 to 2825 (*D*) U2OS or RPE1 cells were transfected twice with 20 nM siRNA targeting GL2 (*control*), SPRYD3 and/or MYCBP2 24 h and 48 h after seeding. 72 h after the first transfection, cells were treated with nocodazole or paclitaxel. Live cell imaging was performed to determine mitotic cell fate of 60 cells. *ns* > 0.05, ∗*p* < 0.05, ∗∗*p* < 0.01, ∗∗∗*p* < 0.001, one-way ANOVA with Dunette *post hoc* test, n = 3.

Since the FBXO45-MYCBP2 E3 ubiquitin ligase plays a significant role in mitotic cell fate decision mechanisms (11), we aimed at understanding more about the function of the E3 ubiquitin ligase in mitosis. We set out to identify new FBXO45-interacting proteins and performed a screen in HEK293T cells based on co-immunoprecipitation with FBXO45 as a bait followed by mass spectrometry analysis (Fig. S1*A*). Our interaction screen identified known FBXO45-binding proteins such as MYCBP2, SKP1, and FBXW7 (11, 20, 21), but also SPRYD3, a SPRY-domain containing and a largely uncharacterized protein (Fig. S1, *A* and *B*) as a new FBXO45-binding partner. We confirmed an interaction between MYCBP2 and SPRYD3 upon co-immunoprecipitation in response to overexpression in HEK293T cells (Fig. 1*A*). SPRYD3 and MYCBP2 also interacted endogenously which suggests that their interaction occurs *in vivo* (Fig. 1*B*). Similar to the SPRY-domain containing protein FBXO45, SPRYD3 harbors SPRY-domains, denoted as SPRY1 and SPRY2, respectively (Fig. S1*C*). SPRYD3 migrates at around 55 kDa (Fig. S1*D*) and binds, similar to FBXO45, to the RING-domain but stronger to the MYC-binding domain of MYCBP2 (Fig. S1*E*), (21). Further truncation of the construct harboring the MYC-binding domain revealed that the minimal binding site of SPRYD3 to MYCBP2 comprises amino acids 2550 to 2825 (Fig. S1*F*). The interaction between SPRYD3 and MYCBP2 occurs through the SPRY2-domain of SPRYD3 (Fig. S1*G*). Although SPRYD3 and MYCBP2 levels are reduced in FBXO45-knock out cells, both proteins can still interact (Fig. S1*H*), suggesting that the interaction is independent of FBXO45. We further showed that interaction between the two proteins is direct using recombinant SPRYD3 and a recombinant MYCBP2 fragment harboring the minimal SPRYD3-binding site (Fig. 1*C*). To determine whether, similar to MYCBP2 (11), SPRYD3 is involved in cell fate decision by inducing mitotic slippage, we analyzed mitotic cell fate by live cell imaging. For the analysis, we treated either U2OS or RPE1 cells with the spindle poisons nocodazole or paclitaxel at concentrations that prevented cell division (22). We find that similar to siRNA-mediated ablation of MYCBP2, treatment with SPRYD3 siRNAs reduced mitotic slippage. Furthermore, double depletion of MYCBP2 and SPRYD3 did not increase mitotic cell death compared to MYCBP2-depleted cells, suggesting that the two proteins work in the same pathway (Fig. 1*D*). Mitotic progression was not affected in cells that were not treated with anti-microtubule drugs in the absence of SRPYD3 (Fig. S2, *A* and *B*). Together, our data suggest that SPRYD3 is a new MYCBP2-interacting protein that together with MYCBP2 may form a new E3 ubiquitin ligase complex to regulate mitotic cell fate decision mechanisms.

### USP11 interacts with the SPRYD3-MYCBP2 E3 ubiquitin ligase and induces mitotic slippage

**Figure 2:**
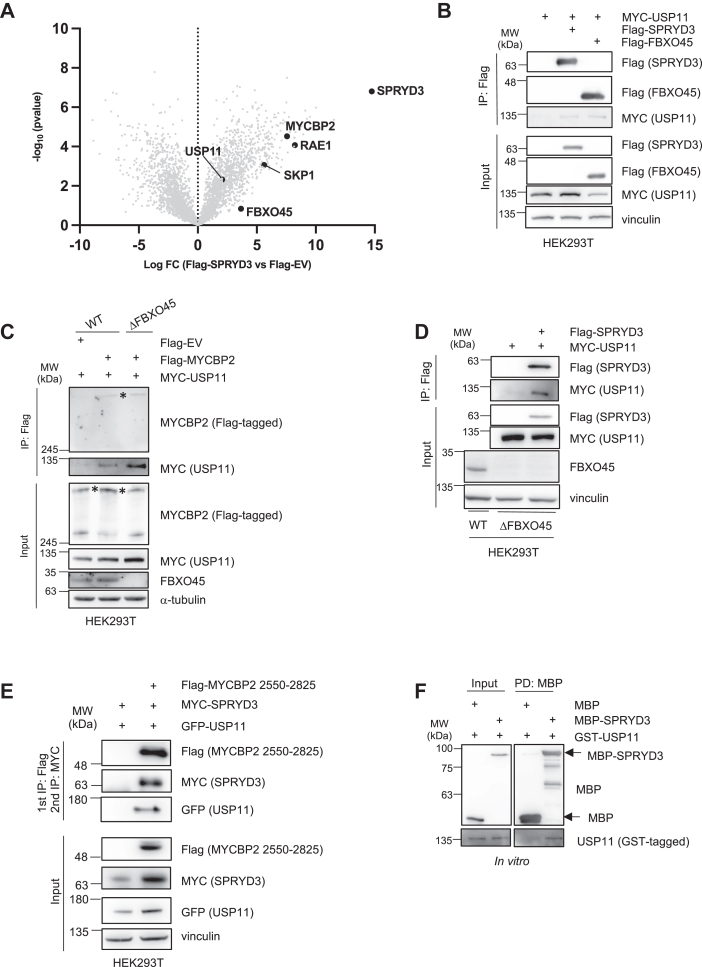
**An interaction screen identifies the DUB USP11 as a new MYCBP2 interacting protein.***A*, Volcano plot demonstrates enrichment of potential SPRYD3 interaction partners identified by combined Flag-IP and MS-analysis approach, n = 4. *B*, HEK293T WT cells were co-transfected with Flag-SPRYD3 or Flag-FBXO45 and MYC-USP11 as indicated and incubated for 24 h. Cells were harvested for Flag-IP. Western blot analysis shows co-precipitation of MYC-USP11 with Flag-tagged FBXO45 or SPRYD3. *C*, Flag-MYCBP2 and MYC-USP11 were ectopically expressed in HEK293T WT or ΔFBXO45 cells for 48 h. Cells were harvested for Flag-IP. Western blot analysis demonstrates interaction of MYC-USP11 with Flag-MYCBP2 in WT or ΔFBXO45 cells. *D*, Flag-SPRYD3 and MYC-USP11 were ectopically expressed in HEK293T ΔFBXO45 cells for 24 h and interaction was analyzed by Western blot. *E*, Flag-MYCBP2 2550 to 2825, MYC-SPRYD3 and GFP-USP11 were co-transfected into HEK 293T WT cells and expressed for 24 h. Cells were treated with 10 μM MG132 5 h prior to harvest. Cells were harvested for Flag-IP. Eluates were used for a consecutive immunoprecipitation against the MYC-tag. Western blot analysis confirms an interaction of all three ectopically expressed proteins. *F*, Recombinant MBP or MBP-SPRYD3 was combined with GST-USP11 to perform a MBP pull-down. Western blot analysis confirms direct interaction of recombinant SPRYD3 and USP11.

**Figure 3:**
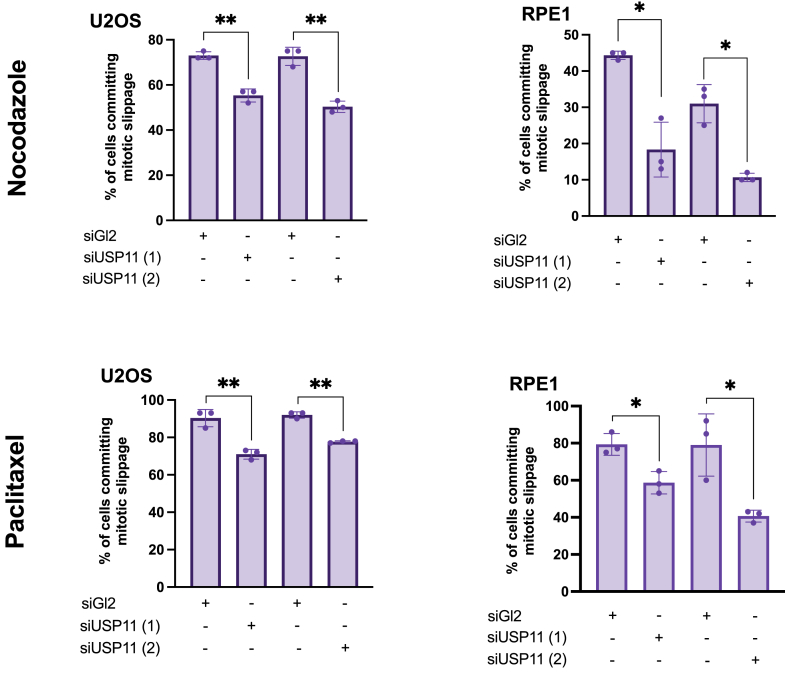
**USP11 promotes mitotic slippage in presence of MT-poisons.** U2OS or RPE1 cells were transfected twice with 20 nM siRNA targeting GL2 (*control*) or USP11 24 h and 48 h after seeding. 72 h after the first transfection, cells were treated with nocodazole or paclitaxel. Live cell imaging was performed to determine mitotic cell fate of 60 cells. ∗*p* < 0.05, ∗∗*p* < 0.01, one-way ANOVA with Dunette *post hoc* test, n = 3.

Similar to FBXO45, SPRYD3 might act as a new substrate receptor for MYCBP2. To identify substrates of SPRYD3-MYCBP2, we performed a screen where we aimed at identifying SPRYD3-binding proteins. SPRYD3 was expressed in HEK293T cells, complexes of SPRYD3 and potential binding partners were immunoprecipitated and subsequent mass spectrometrical analysis was performed. Interestingly, we find the known MYCBP2-interacting proteins RAE1 and FBXO45 in our screen but surprisingly also ubiquitin-specific protease 11 (USP11), a deubiquitinase (Fig. 2*A*) (15, 23). Notably, SPRYD3 was previously also identified in a USP11-interaction screen (24). USP11 is recognized as a tumor promoter and plays a vital role in cell division by regulating microtubule nucleation and facilitating bipolar spindle formation through its deubiquitination activity (24, 25). Based on these well-established functions, we hypothesized that USP11 could affect mitotic cell fate decisions. We confirmed the interaction between SPRYD3 and USP11 (Fig. 2*B*) and MYCBP2 and USP11 (Fig. 2*C*) by co-immunoprecipitation. Binding USP11 to MYCBP2 is independent of the presence of FBXO45 (Fig. 2*C*). In the absence of FBXO45, binding between SPRYD3 and USP11 still occurred (Fig. 2*D*). To assess whether MYCBP2, SPRYD3 and USP11 form one complex, we overexpressed Flag-MYCBP2 2550 to 2825, MYC-SPRYD3 and GFP-USP11 in HEK293T cells and performed two consecutive immunoprecipitations first against the Flag- and consequently against the MYC-tag. GFP-USP11 was present in the final eluate, suggesting that all three proteins are present in the same complex (Fig. 2*E*). A pull-down assay using recombinant SPRYD3 and USP11 showed that the interaction between both of these proteins is direct (Fig. 2*F*). Interestingly, USP11 mainly binds to two different regions of MYCBP2, namely the N-terminal domain harboring an RCC1-like domain and to the MYC-binding domain, which is located in the middle part of the E3-ligase (Fig. S3 and S1*E*). We then analyzed whether USP11 plays a mitotic slippage-inducing role similar to SPRYD3 and MYCBP2. We performed live cell imaging in the absence of USP11 by inducing mitotic arrest in response to treatment with nocodazole or paclitaxel. Using two different cell lines, we find that mitotic slippage was markedly reduced in the absence of USP11 (Fig. 3). Non-drug-treated cells divided normally in the absence of USP11 and did not differ in mitotic timing compared to siRNA controls (Fig. S2, *C* and *D*). Together, these data suggest that SPRYD3-MYCBP2 and USP11 may act in the same pathway to regulate cell fate decision mechanisms.

### SPRYD3-MYCBP2 activity leads to polyubiquitination of USP11

**Figure 4:**
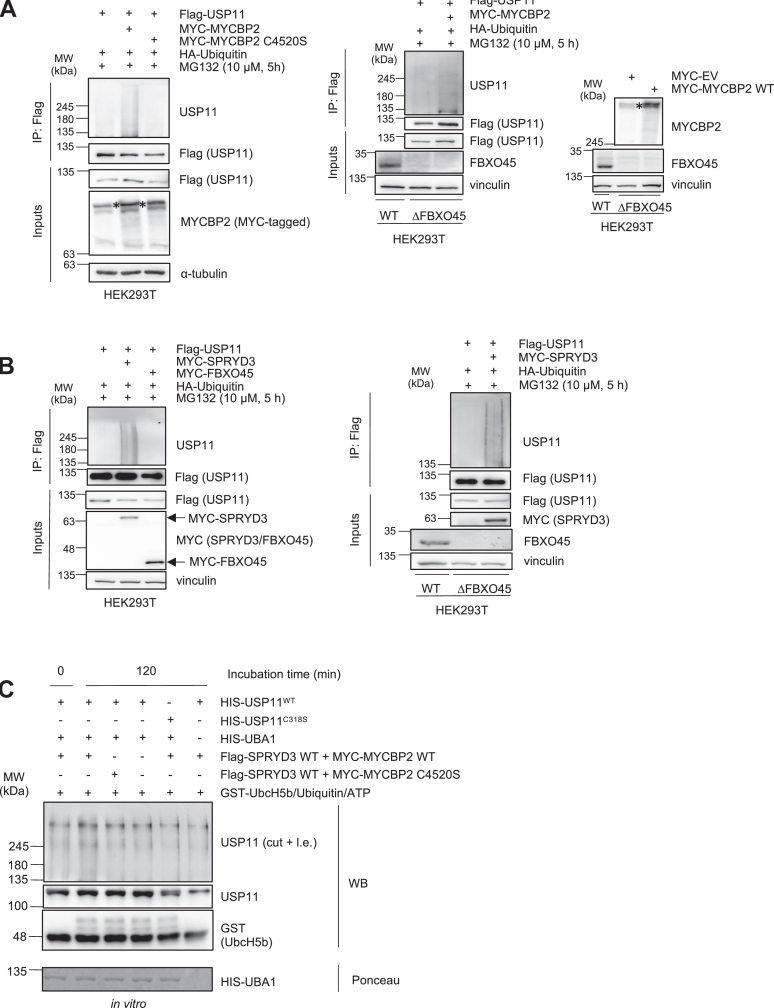
**SPRYD3-MYCBP2 activity leads to ubiquitination of USP11 *in vivo*.***A*, Flag-USP11, MYC-MYCBP2 or MYC-MYCBP2 C4520S and HA-Ubiquitin were co-transfected into HEK293T WT cells (*left*) and Flag-USP11, MYC-MYCBP2 and HA-Ubiquitin were co-transfected into HEK293T ΔFBXO45 cells (*right*) and overexpressed for 48 h. MG132 was added 5 h prior to cell harvest for Flag-IP. Western blot analysis was performed to assess USP11 ubiquitination status. ∗ indicates MYCBP2. *B*, Flag-USP11, MYC-SPRYD3 or MYC-FBXO45 and HA-Ubiquitin were co-transfected into HEK293T WT cells (*left*) and Flag-USP11, MYC-SPRYD3 and HA-Ubiquitin were co-transfected into HEK293T ΔFBXO45 cells (*right*) and overexpressed for 24 h. MG132 was added 5 h prior to cell harvest for Flag-IP. Western blot analysis demonstrates USP11 ubiquitination status in both cell lines. *C*, *in vitro* ubiquitination assay was performed by combining SPRYD3-MYCBP2 E3 ligase complexes precipitated from HEK293T WT cells with recombinant HIS-Uba1, GST-UbcH5b, ubiquitin and ATP as indicated. Ubiquitination reactions were incubated for 0 or 120 min at 30 °C. Ubiquitinated USP11 was detected with endogenous antibody by Western blot analysis. l.e, long exposure; WB, Western blot; Ponceau, Ponceau staining.

FBXO45-MYCBP2 functions as an E3 ubiquitin ligase, and FBXO45 mediates the interaction with substrates (20, 26). Thus, we hypothesized that the SPRYD3-MYCBP2 complex also functions as an E3 ubiquitin ligase with SPRYD3 as a new specificity factor to target USP11 for ubiquitination. To show this, we performed *in vivo* ubiquitination assays. We find that ectopic expression of full-length MYCBP2, but not MYCBP2 C4520S, a catalytically inactive mutant (6), promotes USP11 ubiquitination (Fig. 4*A*). The observed ubiquitination was independent of the presence of FBXO45 (Fig. 4*A*). A C-terminal RING-domain that is critical for the E3 ubiquitin ligase role of *MYCBP2* mediates the ubiquitination modification process of the target protein (5). Co-expression of a fragment harboring only the MYCBP2 RING-domain (MYCBP2 3601–3640) is sufficient to drive ubiquitination of USP11 in the presence and absence of FBXO45 (Fig. S4*A*). Ubiquitination of USP11 was also observed upon overexpression of SPRYD3, but not when FBXO45 was expressed in HEK293T cells (Fig. 4*B*). Similarly, in the absence of FBXO45, ubiquitination of USP11 by SRPYD3 still occurred (Fig. 4*B*). The ability of SPRYD3-MYCBP2 mediating ubiquitination of USP11 as a complex was also shown by the inability of MYCBP2 or SPRYD3 to ubiquitinate USP11 in SPRYD3-or MYCBP2-depleted HEK293T cells, respectively (Fig. S4, *B* and *C*). Ubiquitination of USP11 was reduced upon expression of SPRYD3 mutants lacking either of the two SPRY-domains (Fig. S4*D*). Furthermore, a slight increase in *in vitro* ubiquitination of USP11 was observed in the presence of Uba1 (E1), UbcH5b (E2) and SPRYD3 WT-MYCBP2 WT E3 ligase complex, but not in the presence of SPRYD3 WT-MYCBP2 C4520S complex or absence of the E3 ligase altogether (Fig. 4*C*). Together, these results suggest that USP11 might function as a substrate for SPRYD3-MYCBP2.

### SPRYD3-MYCBP2-mediated ubiquitination of USP11 ensures proper bipolar spindle formation

Protein ubiquitination may modulate protein levels, activity and/or localization of proteins. While lysine-48-linked polyubiquitination is involved in proteolysis of proteins, lysine-63-linked polyubiquitination is shown to have non-proteolytic functions, including chromatin dynamics, kinase and phosphatase activation and signal transduction (27, 28, 29). To find out which ubiquitin-linked chain is present in USP11, K48R or K63R ubiquitin variants were co-expressed with MYCBP2 and USP11. We found that USP11 still showed polyubiquitination in the presence of the K48R ubiquitin variant, but not when K63R was expressed, suggesting that USP11 ubiquitination occurs *via* K63-linked ubiquitin chains (Fig. S5*A*). We further assessed whether SPRYD3-MYCBP2 regulates the protein stability of USP11. To investigate USP11 protein turnover in the presence or absence of SPRYD3, we performed cycloheximide (CHX) chases to stop protein translation. We generated a U2OS cell line where SPRYD3 expression could be induced in response to doxycycline addition. In this setup, USP11 turnover did not differ in the absence or presence of MYC-SPRYD3 under CHX conditions (Fig. S5*B*). In another setup, we performed a CHX chase in response to siRNA-mediated downregulation of SPRYD3, which also did not significantly affect USP11 turnover (Fig. S5*C*). This suggests that SPRYD3-MYCBP2-mediated ubiquitination of USP11 is not related to proteasomal destruction.

**Figure 5:**
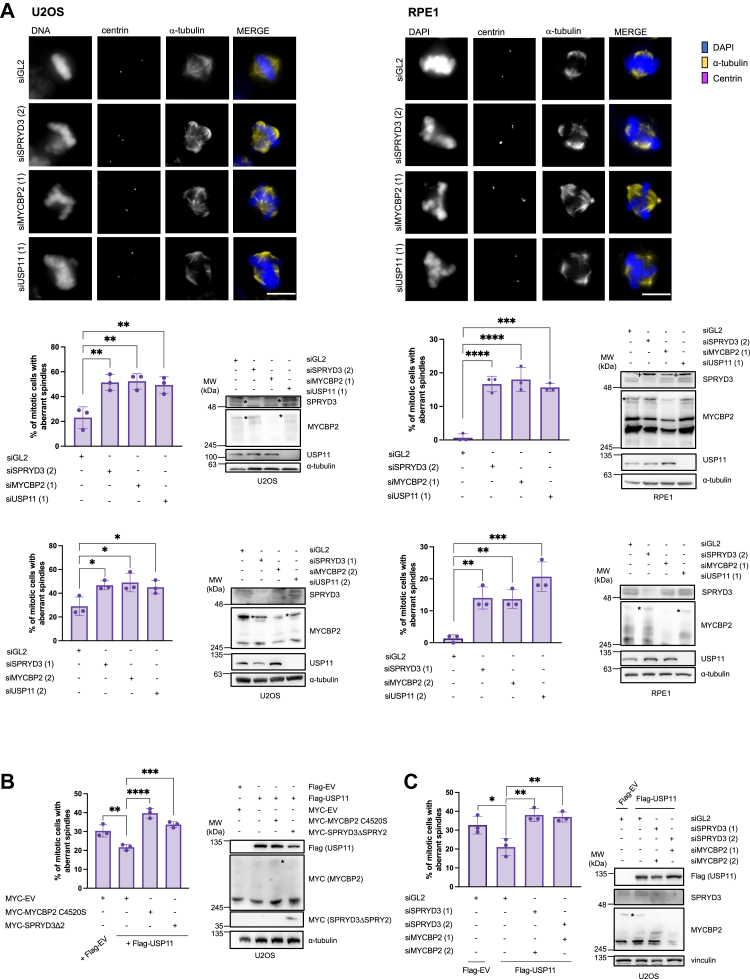
**SPRYD3-MYCBP2 functions in the same pathway as USP11 to promote bipolar spindle formation.***A*, U2OS or RPE1 cells were transfected twice with 50 nM siRNA targeting GL2 (*control*), SPRYD3, MYCBP2 or USP11 using two different siRNAs for each protein of interest 24 h and 48 h after seeding. 48 h after the first transfection, cells were incubated with nocodazole for 24 h. After nocodazole wash-out, cells were treated with MG132 for additional 90 min before fixation and staining (24). For each experiment, 55 (U2OS) or 65 (RPE1) mitotic cells were analyzed for mitotic spindle pole number. Results were subjected to one-way ANOVA with Dunette *post hoc* test, n = 3. ∗*p* < 0.05, *∗∗p* < 0.01, *∗∗∗p* < 0.001, *∗∗∗∗p* < 0.0001. ∗ indicates SPRYD3 or MYCBP2 respectively; + indicates unspecific band. *B*, U2OS cells were co-transfected with Flag-USP11 and MYC-MYCBP2 C4520S or MYC-SPRYD3ΔSPRY2 as indicated. 24 h after transfection, cells were treated with nocodazole and MG132 as described in (*A*). After fixation and staining, 65 mitotic cells were analyzed for mitotic spindle pole number. Results were subjected to one-way ANOVA with Dunette *post hoc* test, n = 3. *∗∗p* < 0.01, *∗∗∗p* < 0.001, *∗∗∗∗p* < 0.0001. ∗ indicates MYC-MYCBP2 C4520S. *C*, U2OS cells were transfected once with a total of 50 nM siRNA targeting GL2 (*control*) or SPRYD3 and MYCBP2 in combination. 24 h after siRNA transfection, cells were transfected with Flag-EV or Flag-USP11 as indicated. 48 h after siRNA transfection, cells were incubated with nocodazole for further 24 h and treated with MG132 as described in (*A*). For each experiment, 65 mitotic cells were analyzed for mitotic spindle pole number. Results were subjected to one-way ANOVA with Dunette *post hoc* test, n = 3. ∗*p* < 0.05, *∗∗p* < 0.01. ∗ indicates MYCBP2.

Cell fate of mitotic cells is controlled by spindle assembly. Deficient spindle assembly results in mitotic catastrophe leading to cell death to maintain cellular homeostasis. Previous data reveal that USP11 affects bipolar spindle formation by deubiquitinating RAE1 at the mitotic spindle (24). In fact, depletion of USP11 affects bipolar spindle formation leading to a multipolar spindle phenotype (Fig. 5*A*) (24). If both MYCBP2 and SPRYD3 regulate USP11 function, it is conceivable that these proteins might also play a role in bipolar spindle formation. We find that either siRNA-mediated depletion of MYCBP2 or SPRYD3 leads to a similar multipolar spindle phenotype as shown for USP11, suggesting that USP11 and SPRYD3-MYCBP2 might collaborate to regulate this process (Fig. 5*A*). To test this hypothesis, we assessed the ability of USP11 to promote bipolar spindle formation in presence of an ubiquitination deficient SPRYD3-MYCBP2 complex. Ectopic expression of USP11 reduced the multipolar spindle phenotype, while co-expression of ubiquitination deficient MYCBP2 or deletion mutant SPRYD3ΔSPRY2, which cannot recruit MYCBP2, reversed this effect (Fig. 5*B*). We obtained similar results after downregulation of SPRYD3-MYCBP2 using siRNAs against both proteins (Fig. 5*C*). This suggests that SPRYD3-MYCBP2-dependent ubiquitination of USP11 might be important for its function in bipolar spindle formation.

**Figure 6:**
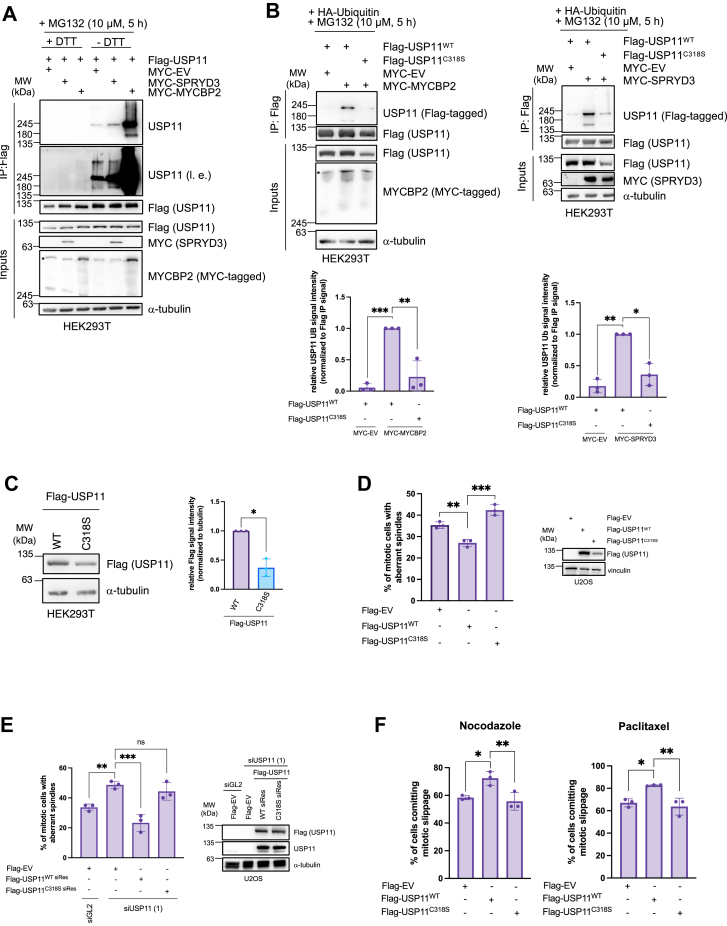
**SPRYD3-MYCBP2-mediated ubiquitylation of USP11^C318^ promotes bipolar spindle formation and mitotic slippage.***A*, HEK293T WT cells were co-transfected with Flag-USP11, MYC-MYCBP2 or MYC-SPRYD3 and HA-Ubiquitin as indicated and incubated for 24 h. Cells were harvested for Flag-IP. IP-samples were denatured in 2x Laemmli buffer ± DTT. Western blot analysis demonstrates USP11 ubiquitination status under ± DTT conditions. ∗ indicates MYCBP2. *B*, Flag-USP11^WT^ or Flag-USP11^C318S^, MYC-MYCBP2 or MYC-SPRYD3 und HA-Ubiquitin were ectopically expressed as indicated in HEK293T WT cells for 24 h. Cells were harvested for Flag-IP. IP-samples were denatured in 2x Laemmli buffer without DTT. Western blot analysis demonstrates USP11 ubiquitination status under -DTT conditions. USP11 ubiquitination status was quantified using Western blot signals after normalization with the respective Flag-IP signal. Flag-USP11^WT^ was used as 100% reference. Relative values were log-transformed to obtain parametric distribution and applied to one sample t and Wilcoxon test, n = 3. ∗*p* < 0.05, ∗∗*p* < 0.01, ∗∗∗*p* < 0.001. ∗ indicates MYCBP2. *C*, HEK293T WT cells were transfected with Flag-USP11^WT^ or Flag-USP11^C318S^ and incubated for 24 h. Cells were harvested for Western blot analysis. Flag-USP11 protein levels were quantified using Western blot signals after α-tubulin-normalization. Flag-USP11^WT^ was used as reference. Relative values were log-transformed to obtain parametric distribution and applied to one sample t and Wilcoxon test, n = 3. ∗*p* < 0.05. *D*, U2OS cells were transfected with Flag-USP11^WT^ or Flag-USP11^C318S^ as indicated. Cells were treated with nocodazole for 24 h and MG132 for additional 90 min prior to harvest (24). Subsequent to fixation and staining, 65 mitotic cells per condition were analyzed for mitotic spindle pole number. Results were subjected to one-way ANOVA with Dunette *post hoc* test, n = 3. *∗∗p* < 0.01, *∗∗∗p* < 0.001. *E*, U2OS cells were transfected twice with 50 nM siRNA targeting GL2 (*control*) or USP11 24 h and 30 h after seeding. 24 h after the first siRNA transfection, U2OS cells were transfected with Flag-USP11^WT siRes^ or Flag-USP11^C318S siRes^ as indicated. After 24 h of incubation, cells were treated with nocodazole and MG132 as described in (*D*). Subsequent to fixation and staining, 65 mitotic cells per condition were analyzed for mitotic spindle pole number. Results were subjected to one-way ANOVA with Dunette *post hoc* test, n = 3. *ns* > 0.05, ∗∗*p* < 0.01, ∗∗∗*p* < 0.001. *F*, U2OS cells were transfected with Flag-USP11^WT^ or Flag-USP11^C318S^ as indicated and treated with nocodazole or paclitaxel 48 h after transfection. Live cell imaging was performed to determine mitotic cell fate of 60 cells. ∗*p* < 0.05, ∗∗*p* < 0.01, one-way ANOVA with Dunette *post hoc* test, n = 3. l.e., longer exposure; siRes, siRNA-resistant.

Since MYCBP2 does not primarily target lysine residues for ubiquitination, we assessed on which amino acid USP11 is ubiquitinated after expression of SPRYD3-MYCBP2. Thus, we tested the ubiquitination level of USP11 in the presence or absence of DTT. This reducing agent can break oxy-ester- but specifically dissolves thioester-bonds between ubiquitin and cysteine residues, leading to the formation of un-ubiquitinated proteins with a lower molecular mass. As shown in Figure 6*A*, ubiquitination levels of USP11 were high in the absence of DTT. Based on this observation, it is possible that ubiquitination may occur on a cysteine residue. One predominant cysteine residue in USP11 is the catalytic C318 (15, 30). To test the hypothesis whether USP11^C318^ is ubiquitinated by SPRYD3-MYCBP2, we generated a catalytically inactive USP11^C318S^ mutant. We noted that this mutant is less stable than USP11^WT^ (Fig. 6*C*). While SPRYD3-MYCBP2-dependent ubiquitination was observed on USP11^WT^, a significant reduction in ubiquitination was noted in ubiquitination assays performed under non-reducing conditions using the USP11^C318S^ mutant as a substrate (Fig. 6*B*). We also included the USP11^C318S^ mutant in an *in vitro* ubiquitination assay and found that it was less ubiquitinated compared to USP11^WT^ (Fig. 4*C*). However, based on our results, we cannot exclude that SPRYD3-MYCBP2 also affects other ubiquitination sites in USP11.

To assess whether USP11 activity plays a role in bipolar spindle formation, we expressed USP11^WT^ or the USP11^C318S^ mutant in cells and found that while USP11^WT^ could reduce the multipolar spindle phenotype, this was not the case when the USP11^C318S^ mutant was expressed (Fig. 6*D*). Furthermore, we show that in USP11-depleted cells, the aberrant spindle phenotype can be rescued by expressing a USP11^WT^ siRNA-resistant mutant, but not upon USP11^C318S^ siRNA-resistant mutant expression, suggesting that the catalytic activity of USP11 is required for bipolar spindle formation (Fig. 6*E*). Moreover, we find that in cells treated with nocodazole or paclitaxel, the rate of mitotic slippage was reduced upon expression of USP11^C318S^ (Fig. 6*F*), which confirms that functional bipolar spindle formation promotes mitotic slippage in arrested cells.

Together, our data show that SPRYD3-MYCBP2 activity results in ubiquitination of the DUB USP11 on C318, probably leading to its activation to regulate bipolar spindle formation in mitosis.

## Discussion

Our investigation on the interaction network of the E3 ligase MYCBP2 highlights the importance of the interplay between DUBs and E3 ligases in understanding regulatory intricacies, particularly in the regulation of mitotic cell fate. This study aimed to unravel the pathways underlying MYCBP2 function in proper bipolar spindle formation, while investigating its regulation by substrate adaptors and downstream mechanisms.

Ubiquitin E3 ligases are often found to interact with DUBs, where the E3 ligase can activate the DUB *via* ubiquitination, while the DUB can, in turn, stabilize the E3 ligase through deubiquitination (31). For instance, it has been previously suggested that the interaction between USP7 (HAUSP) and the p53 E3 ligase MDM2 helps to stabilize MDM2, leading to lower p53 protein levels (32). In addition, K63-ubiquitinated USP7 (HAUSP) deubiquitinates the hypoxia-inducible factor HIF-1α (14). Our research has revealed that the E3 ligase MYCBP2, together with a new substrate adaptor SPRYD3, does not function as a destabilizer of USP11 protein levels, but as a modulator of USP11 activity in the regulation of bipolar spindle formation. We have shown that SPRYD3-MYCBP2 expression affects the catalytic cysteine residue, C318, in USP11 to probably regulate mitotic spindle formation. Historically, research has primarily focused on the conjugation of ubiquitin to lysine residues on substrates. Ubiquitination can also take place on cysteine, serine, and threonine residues, in addition to N-terminal amino groups of proteins (33, 34). Cysteines play crucial roles in the ubiquitination cascade. The E1 and E2 enzymes, along with several classes of E3 enzymes, bind to ubiquitin through a thioester linkage to cysteine. Thioesters are significantly more reactive than amides, making them much more prone to nucleophilic transfer (34). MYCPB2 has been previously shown to generate oxy-ester linkages. The RING-domain of MYCBP2 interacts with the E2 enzyme to promote the transfer of ubiquitin to its tandem cysteine domain, where the ubiquitin is subsequently passed to the tandem cysteines and finally transferred to the substrate (5, 6). MYCBP2 creates non-lysine linkages, which implies that it might target USP11 on cysteine C318 (Figs. 4*C* and 6*B*). However, further studies will be necessary to show that USP11 is a direct substrate of SPRYD3-MYCBP2. We further demonstrate that expression of the catalytically inactive USP11^C318S^ mutant (15) impairs mitotic spindle formation, leading to aberrant mitotic spindles (6D and E). It is conceivable that mitotic cell fate decisions require quick transient modifications, which could be facilitated through thio-esters as they can form rapidly (34).

Overall, our work unravels a new pathway underlying mitotic spindle formation involving the activation of the deubiquitination activity of USP11 by the E3 ligase SPRYD3-MYCBP2, which might affect critical downstream substrates of USP11. One of its substrates is the microtubule-binding protein tau, which promotes the assembly and stabilization of microtubules, thus affecting cell division. Future studies will be needed to unravel the pathway underlying the inactivation of USP11 in mitotic cell fate regulation.

## Experimental procedures

### Cell lines and cell culture

HEK293T WT (Cat#: ACC 635; DSMZ, Braunschweig), HEK293T ΔFBXO45 (Hoffmann lab, DKFZ Heidelberg), U2OS (HTB-96; ATCC) and U2OS Flp-In-T-REX MYC-SPRYD3 cells were cultured in Dulbecco’s modified Eagle’s medium (Cat#: 41965-039; Gibco). hTERT RPE1 (CRL-4000; ATCC) were cultured in Dulbecco’s Modified Eagle’s Medium/Nutrient Mixture F-12 Ham (Cat#: D8900; Sigma-Aldrich). Both growth media were supplemented with 10% fetal bovine serum (Cat#: 10270-106; Gibco) and 1% penicillin-streptomycin (Cat#: P0781; Sigma-Aldrich). All cell lines were maintained in a humified CO_2_ incubator at 37 °C and 5% CO_2_. The U2OS Flp-In-T-REX MYC-SPRYD3 stable cell line was generated using the T-Rex system according to manufacturer’s orders (Life Technologies). The 26S proteasome was blocked with MG132 (Cat#: C2211; Sigma-Aldrich) for at least 5 h prior to harvest. Cell lines were regularly tested for *mycoplasma* contamination and cell culture work was performed under sterile conditions. Cell line identities were periodically tested and verified by authentication (Multiplexion).

### Plasmids, cloning and mutagenesis

pGM-Flag-SPRYD3 was a kind gift from Dr Goedele Maertens (Imperial College London). pCMV-3Tag-1A-Flag-SPRYD3 or pCMV-3Tag-2A-MYC-SPRYD3 were generated by subcloning using EcoRI HF and HINDIII HF. Flag- and MYC-tagged SPRYD3ΔSPRY1 and -SPRYD3ΔSPRY2 constructs were generated by site-directed mutagenesis (35) using restriction enzymes EcoRI HF and HINDIII HF. pMal-c2-MBP-SPRYD3 was generated by subcloning using EcoRI HF and HINDIII HF. pcDNA5-FRT-TO-MYC-SPRYD3 was generated by subcloning using HINDIII HF and XhoI. pCMV-MYC-MYCBP2 was purchased from Addgene (plasmid #42570), and Flag-tagged MYCBP2 constructs 1 to 1050, 1051 to 1950, 1951 to 2950, 2951 to 3600 and 3601 to 4640 have been described previously (11). pCMV-3Tag-1A-Flag-MYCBP2 1951 to 2400 was provided by Kai Richter (Hoffmann Lab, DKFZ). Flag-tagged MYCBP2 truncations 2400 to 2700, 2400 to 2550 and 2551 to 2700 were generated by site-directed mutagenesis (35) and subcloning using EcoRI HF and HINDIII HF. Flag-tagged MYCBP2 truncations 2400 to 2950, 2700 to 2950 and 2550 to 2825 were generated by site-directed mutagenesis (35) and subcloning using BamHI HF and XhoI. pQCXIZ-Flag-MYCBP2 and pCMV-3Tag-2A-MYC-MYCBP2 C4520S were provided by Kai Richter (Hoffman Lab, DKFZ). pGEX4T1-GST-MYCBP2 2550 to 2825 was generated by subcloning using BamHI HF and XhoI. pCMV-3Tag-1A-Flag-FBXO45 and pCMV-3Tag-2A-MYC-FBXO45 have been described previously (11). pDEST-LTR-N-Flag-HA-IRES-puro-USP11 was purchased from Addgene (plasmid #22566). pCMV-3Tag-1A-Flag-USP11^WT^, pCMV-3Tag-2A-MYC-USP11^WT^ and pEGFP-C1-USP11^WT^ were generated by subcloning using XhoI and EcoRI HF. pCMV-3Tag-1A-Flag-USP11C^318S^ was generated *via* site-directed mutagenesis (35) and subcloning using EcoRI HF and HINDIII HF. siRNA-resistant (siRes) plasmids Flag-USP11^WT siRes^ and Flag-USP11^C318S siRes^ were generated by changing every third base pair of the siUSP11 (1) siRNA target sequence GGACCGTGATGATATCTTC (30) into AGATCGAGACGACATATTT. A fragment containing this siRNA-resistant sequence was synthesized by GenScript and subcloned into the original plasmids using restriction enzymes ScaI and XhoI (Flag-USP11^WT siRes^) or ScaI and HINDIII HF (Flag-USP11^C318S siRes^). pGEX4T1-GST- USP11 was generated by subcloning using EcoRI HF and XhoI. pET28a(+)-HIS-USP11^WT^ and pET28a(+)-HIS-USP11^C318S^ were generated by subcloning using EcoRI HF and XhoI (USP11^WT^) or EcoRI HF and HINDIII HF (USP11^C318S^). pPK-CMV-HA-UbiquitinC, pPK-CMV-HA-UbiquitinC K48R and pPK-CMV-HA-UbiquitinC K63R were kind gifts from Frank Rösl (DKFZ Heidelberg). pSG5-His-myc-Ubiquitin was a kind gift from Prof. Martin Scheffner (University of Konstanz).

### Transient plasmid and siRNA transfections

HEK293T WT and HEK293T ΔFBXO45 cells were transiently transfected with plasmid DNA using polyethylenimine at a final concentration of 5 μg/ml and harvested after 24 h or 48 h, if not stated otherwise. For experiments with combined siRNA treatment and plasmid DNA transfection, HEK293T WT cells were transfected twice with 20 nM siRNA using Lipofectamine RNAiMAX (Cat#: 13778150; Invitrogen) according to the manufacturer’s orders. 48 h after the first siRNA transfection, cells were transfected with plasmid DNA as described above and harvested after 72 h of the first siRNA transfection. U2OS and RPE1 cells were transfected twice with 20 or 50 nM siRNA using Lipofectamine RNAiMAX according to the manufacturer’s orders. U2OS cells were transfected with siRNA, plasmid DNA, or both combined using [_lrl_2000] (Cat#: 11668019; Invitrogen) according to the manufacturer’s orders.

Sequences of siRNA used in experiments are stated below:

siGL2 (firefly luciferase; control): 5′ -CGUACGCGGAAUACUUCGAdTdT- 3′ (Eurofins)

siSPRYD3 (1): 5′ -AUGGAGAUACUUUAAGUUA- 3′ (Ambion, Silencer Select)

siSPRYD3 (2): 5′ – UCAUGAUGGUGGACAGUUA- 3′ (Ambion, Silencer Select)

siUSP11 (1): 5′ -GGACCGUGAUGAUAUCUUC- 3′ (30)

siUSP11 (2): 5′ -GAAGAAGCGUUACUAUGAC- 3′ (30)

siMYCBP2 (1): 5′ -CCCGAGAUCUUGGGAAUAAtt- 3′ (Eurofins)

siMYCBP2 (2): 5′ -GGAAGUAGUUCUGUUAUUUtt- 3′ (Dharmacon)

### Cell lysis, co-immunoprecipitation, and Western blot analysis

Cell lysates were prepared as described previously (36). Briefly, cells were lysed with RIPA buffer (50 mM Tris–HCl, pH 7.4, 1% NP-40, 0.5% sodium deoxycholate, 0.1% SDS, 150 mM NaCl, 2 mM EDTA, and 50 mM NaF) supplemented freshly with 1 mM DTT, 10 μg/ml l-1-tosylamido-2-phenylethyl chloromethyl ketone, 5 μg/ml tosyl lysyl chloromethyl ketone, 0.1 mM Na_3_VO_4_, 1 μg/ml aprotinin, 1 μg/ml leupeptin, and 10 μg/ml trypsin inhibitor from soybean for Western blot analysis. For IPs, cells were lysed with NP-40 buffer (40 mM Tris–HCl, pH 7.5, 150 mM NaCl, 5 mM EDTA, 10 mM β-glycerophosphate, 5 mM NaF, and 0.5% NP-40) supplemented with 1 mM DTT, 10 μg/ml l-1-tosylamido-2-phenylethyl chloromethyl ketone, 5 μg/ml tosyl lysyl chloromethyl ketone, 0.1 mM Na_3_VO_4_, 1 μg/ml aprotinin, 1 μg/ml leupeptin, and 10 μg/ml trypsin inhibitor from soybean. For SDS-PAGE analysis, 50 μl of protein extracts were mixed with 2x Laemmli buffer and incubated at 95 °C for 5 min to be used as inputs. Flag-tagged proteins were immunoprecipitated with Flag-M2 beads (Cat#.: A2220; Sigma-Aldrich) by incubation at 4 °C overnight. Bound Flag-tagged proteins were eluted by incubation with 3x Flag peptide (Cat#: A36805; Thermo Fisher Scientific) (final concentration 150 ng/ml). Eluates were mixed with 2x Laemmli buffer and boiled at 95 °C for 5 min. Whole protein-extracts and immunoprecipitations were analyzed by SDS-PAGE and Western blot. For consecutive co-immunoprecipitation, protein lysates were used to perform a Flag-immunoprecipitation (Flag-IP) as described above. Elutions were used for consecutive immunoprecipitation against MYC-tag. 4 μg of anti-MYC antibody (Cat#: sc-40, 9E10; Santa Cruz Biotechnologies) was added overnight at 4 °C, rotating on a wheel. Simultaneously, G-sepharose beads (mouse) were equilibrated overnight in 0.1% BSA in 1x PBS at 4 °C. Consequently, G-sepharose beads were added to MYC-immunoprecipitations and incubated for 2 h at 4 °C rotating on a wheel. Samples were washed with NP-40 buffer and protein complexes were eluted by incubation with 50 μl 2x Laemmli buffer at 95 °C for 5 min. For endogenous immunoprecipitation of SPRYD3, HEK293T WT cell lysates treated with 10 μM MG132 for 5 h prior to harvest were incubated with 4 μg of anti-SPRYD3 antibody (Cat#: NBP1-74270; Novus) or anti-IgG (rabbit) antibody overnight. Immunoprecipitations were incubated with protein A sepharose beads (rabbit) at 4 °C for 2 h and consequently washed with NP-40 buffer adjusted to 400 mM NaCl. Proteins were eluted from beads by incubation with 2x Laemmli buffer at 95 °C for 5 min.

MYCBP2 protein levels were analyzed by a Tris-acetate gel and buffer system as described in (37). MYCBP2 protein transfer onto nitrocellulose (Cat#: 10600013; Cytiva) after SDS-PAGE was executed at 0.2 mA for 2 h at 4 °C. Detection of all proteins was executed by chemiluminescence using the Immobilon western HRP Substrate Luminol Reagent and HRP Substrate Peroxide Solution (Cat#: WBKLS0500; Merck) according to the manufacturer’s orders.

### Cycloheximide (CHX) chase assay

To determine USP11 protein turnover in the presence of MYC-SPRYD3, U2OS T-REX MYC-SPRYD3 clone (8) was treated with 2 μg/ml doxycycline (CAS#: 10592-13-9; Sigma-Aldrich) for 72 h to induce MYC-SPRYD3 expression. 100 μg/ml cycloheximide (CHX) (Cat#: sc-3508; Santa Cruz Biotechnologies) was added to cells for the indicated time points in order to block protein synthesis. To determine USP11 protein turnover in the absence of SPRYD3, U2OS cells were transfected twice with 20 nM siRNA 24 h and 48 h after seeding and consequently treated as described above. Protein lysates were analyzed by SDS-PAGE and Western blot.

### MBP pull-down assay

Recombinant MBP-SPRYD3, GST-MYCBP2 2550 to 2825 or GST-USP11 were expressed in *E. coli* Rosetta (DE3) and purified using Amylose resin (Cat#: E8021S; New England BioLabs) or Glutathione Sepharose^Tm^ 4B (Cat#: GE17-0756-05; Merck). To prove a direct interaction between SPRYD3 and MYCBP2 2550 to 2825 or SPRYD3 and USP11, 10 μg of MBP-SPRYD3 or equimolar amount of empty MBP were mixed with 10 μg GST-MYCBP2 2550 to 2825 or GST-USP11 in NP-40 buffer. The recombinant protein mixtures were rotated on a wheel at 4 °C for 1 h. Amylose resin (Cat#: E8021S; New England BioLabs) was added to recombinant protein mixture and incubated for 2 h at 4 °C. Resin with bound proteins was washed five times with NP-40 buffer and protein complexes were eluted by incubation with 30 μl of 2x Laemmli buffer for 5 min at 95 °C. Samples were analyzed by SDS-PAGE and Western blot.

### *In vivo* ubiquitination assay

HEK293T WT or ΔFBXO45 cells were transfected with indicated plasmids and incubated for 24 h or 48 h. Cells were treated for additional 5 h with 10 μM proteasome inhibitor MG132 prior to harvest. For Flag-immunoprecipitation, cells were lysed in NP-40 buffer supplemented with 20 mM N-ethylmaleimide (Cat#: E3876; Sigma-Aldrich). Flag-immunoprecipitations were performed as described previously. For ubiquitination assays performed under denaturing conditions, HEK293T WT or ΔFBXO45 were lysed with 8 M Urea supplemented with 0.1 M phosphate buffer pH 8.0, 20 mM N-ethylmaleimide, 30 mM imidazole and 1M DTT, 10 μg/ml l-1-tosylamido-2-phenylethyl chloromethyl ketone, 5 μg/ml tosyl lysyl chloromethyl ketone, 0.1 mM Na_3_VO_4_, 1 μg/ml aprotinin, 1 μg/ml leupeptin, 10 μg/ml trypsin inhibitor from soybean. Cells were sonicated using a Branson Sonifier 250. Protein lysates were incubated with Ni-NTA agarose (Cat#: 30210; Qiagen) for 3 h at room temperature. Bound proteins were washed with 8 M urea buffer supplemented with 0.1% Triton X-100. Proteins were eluted with 2x Laemmli buffer supplemented with 200 mM imidazole and incubation for 10 min at room temperature. Samples were denatured at 95 °C for 5 min. Whole protein extracts and pull-downs were analyzed by SDS-PAGE and Western blot.

### *In vitro* ubiquitination assay

HIS-USP11^WT^ or HIS-USP11^C318S^ were recombinantly expressed and purified from *E. coli* Rosetta (DE3) using Ni-NTA agarose. Assays were performed as described by (36), but concentration of enzymes, incubation time and temperature were slightly changed. Recombinant HIS-UBA1, GST-UbcH5b and ubiquitin were kind gifts from Frauke Melchior (Heidelberg University). ATP (Cat#: R0441) was purchased from Thermo Fisher Scientific. Briefly, E3 ligase complexes were overexpressed in HEK293T WT cells for 24 h and immunoprecipitated using Flag-M2 beads as described previously. Bound protein complexes were eluted from beads in ubiquitin assay buffer (50 mM Tris-HCl, pH 7.5, 100 mM NaCl, 10 mM MgCl_2_, 0.05%,1 mM DTT, 0.1 mM Na_3_VO_4_, 1 μg/ml aprotinin, 1 μg/ml leupeptin,10 μg/ml trypsin inhibitor from soybean and 1 mg/ml BSA with final concentration of 1:20). For *in vitro* ubiquitination reactions, 10 μl of eluted E3 ligase complex, 500 nM HIS-USP11^WT^ or HIS-USP11^C318S^, 170 nM HIS-UBA1, 30 μM ubiquitin, 250 nM GST-UbcH5b and 5 mM ATP were combined in 20 μl total volume of ubiquitin assay buffer. Ubiquitination reactions were incubated for 0 or 120 min at 30 °C. Termination of reaction was achieved by addition of 2x NuPAGE LDS buffer (Cat#: NP0007; Thermo Fisher Scientific) and denaturation at 72 °C for 10 min. Proteins were resolved by SDS-PAGE and Western blot.

### Antibodies

Following antibodies were used for protein detection in Western blot analysis: anti-SPRYD3 (Cat#: NBP1-74270) was purchased from Novus; anti-MYCBP2 (Cat#: ab86078) was purchased from Abcam; anti-GST (Cat#: sc-459, Z-5), anti-USP11 (Cat#: sc-365528, C-6), anti-MYC (Cat#: sc-40, 9E10) and anti-SKP1 (Cat#: sc7163, H-163) were purchased from Santa Cruz Biotechnologies; anti-Flag (catalog no.: F3165), anti-α-tubulin (Cat#: T5168; IgG_1_) and anti-vinculin (Cat#: V9131, h-VIN1) were purchased from Sigma-Aldrich; anti-FBXW7 (Cat#: A301-720) was purchased from Bethyl; anti-RAE1 was a kind gift of Michael D. Blower[_blower_et_al_2005]; anti-FBXO45 was provided by the Ingrid Hoffmann Laboratory at DKFZ; anti-centrin (Cat#: 04-1624, 20H5; IgG_2a_) was purchased from Merck; anti-GFP (Cat#: 50430-2-AP) was purchased from Proteintech; anti-mouse IgG HRP from goat was purchased from Novus; anti-rabbit IgG HRP from donkey was purchased from Jackson laboratories; secondary antibodies for immunofluorescence anti-mouse IgG_1_ Alexa 488 (goat) (Cat#: A21121) and anti-mouse IgG_2a_ Alexa 594 (goat) (Cat#: A-21135) were purchased from Invitrogen.

### Immunofluorescence microscopy

U2OS or RPE1 cells were seeded on cover slips and transfected with siRNA, plasmid DNA or both combined as described previously. 24 h or 48 h after the first siRNA or plasmid DNA transfection, 200 ng/ml nocodazole was added for an additional 24 h. Nocodazole was carefully removed and cells were treated for an additional 90 min with 5 μM MG132 (24). Cells were fixed with ice-cold methanol for 10 min at −20 °C. Primary (anti-α-tubulin: 1:2000; anti-centrin: 1:500) and secondary antibody (1:2000) incubations were performed at room temperature for 1 h each. Cover slips were mounted onto glass microscope slides using ProLong Gold Antifade with DAPI (Cat#: P36935; Life Technologies) for DNA visualization. Images were taken using a 63x/1.4 Oil Pln Apo DICII objective on a Zeiss Cell Observer.Z1 inverted microscope (AxioCam MRm camera system). Images were analyzed using the Fiji software (38).

### Live cell imaging

For mitotic cell fate analysis, live cell imaging was performed. U2OS or RPE1 cells were transfected with 20 nM siRNA as described previously. Cells were seeded into 8-well dish chambers (Cat#: 94.6170.802; Sarstedt) and treated with nocodazole (250 ng/ml for U2OS; 3.1 μM for RPE1) or paclitaxel (1 μM for U2OS; 5 μM for RPE1) 72 h after the first transfection. Consequently, cells were placed in a microscopy incubation chamber at 5% CO_2_ and 37 °C. For analysis of unperturbed mitosis and mitotic timings, the same procedure was executed without microtubule poison treatment. Cells were monitored using a 10 x/0.3 EC PlnN Ph1 DICI objective on a Zeiss Cell Observer.Z1 inverted microscope (AxioCam MRm camera system). Every 10 min for up to 60 h, phase-contrast images of multiple positions were taken using the Zeiss ZEN blue software. The Fiji software was used to analyze mitotic cell fates or unperturbed mitosis (38). Cell morphology was used to determine cell death, whereas mitotic slippage was defined by mitotic exit without previous division. Normal cell division is counted as unperturbed mitosis. To determine mitotic timings of untreated dividing cells, rounded up morphology was used to recognize the start of mitosis and cytokinesis was set as the end point.

### Mass spectrometry analysis of Flag-FBXO45 and Flag-SPRYD3 interaction partners

For identification of potential interactors and/or substrates of FBXO45 or SPRYD3, Flag-EV (control), Flag-FBXO45, Flag-FBXO45ΔSPRY or Flag-SPRYD3 was transfected into HEK293T WT cells and expressed for 30 h. Cells were harvested and lysed with NP-40 buffer. Flag-IP was performed as described previously with following variations: 20 to 25 mg/ml of protein lysate was added to 80 μl of Flag-M2 beads (Cat#.: A2220; Sigma-Aldrich) to immunoprecipitate Flag-tagged proteins overnight. Immunoprecipitated proteins were eluted from Flag-M2 beads using 70 μl of 3x Flag-peptide at a final concentration of 500 ng/ml. Eluates were boiled at 95 °C for 5 min in 2x Laemmli buffer. In total, four biological replicates were performed for each set. All replicates were handed over to the DKFZ Proteomics Facility, which performed the MS-analysis and statistics and provided the protocol. Briefly, IP eluates were loaded onto an SDS-PAGE and run 0.5 cm into the gel. The entire piece was cut out and digested with trypsin (39) and adapted to a DigestPro MSi robotic system (INTAVIS Bioanalytical Instruments AG). The LC-MS/MS analysis was executed using an Ultimate 3000 UPLC system (Thermo Fisher Scientific) directly connected to an Orbitrap Exploris 480 mass spectrometer for a total of 120 min. Peptides were desalted online on a trapping cartridge (Acclaim PepMap300 C18, 5 μm, 300Å wide pore; Thermo Fisher Scientific) for 3 min using 30 μl/min flow of 0,05% TFA in water. The analytical multistep gradient (300 nl/min) was performed using a nanoEase MZ Peptide analytical column (300Å, 1.7 μm, 75 μm × 200 mm, Waters) using solvent A (0.1% formic acid in water) and solvent B (0.1% formic acid in acetonitrile). For 102 min the concentration of B was linearly ramped from 4% to 30%, followed by a quick ramp to 78%, after 2 minutes the concentration of B was lowered to 2% and a 10 min equilibration step appended. Eluting peptides were analyzed in the mass spectrometer using data dependent acquisition (40) mode. A full scan at 120 k resolution (380–1400 m/z, 300% AGC target, 45 ms maxIT) was followed by up to 2 s of MS/MS scans. Peptide features were isolated with a window of 1.4 m/z, fragmented using 26% NCE. Fragment spectra were recorded at 15 k resolution (100% AGC target, 54 ms maxIT). Unassigned and singly charged eluting features were excluded from fragmentation and dynamic exclusion was set to 30 s. Each sample was followed by a wash run (40 min) to minimize carry-over between samples. Instrument performance throughout the course of the measurement was monitored by regular (approx. one per 48 h) injections of a standard sample and an in-house shiny application. Data analysis was carried out by MaxQuant[_tyanova_et_al_2018] using an organism specific database extracted from Uniprot.org (human reference database, containing 74,811 unique entries from 27th of February 2020). Settings were left at default with the following adaptations. Match between runs (MBR) was enabled to transfer peptide identifications across RAW files based on accurate retention time and m/z. Fractions were set in a way that MBR was only performed within replicates. Label free quantification (LFQ) was enabled with default settings. Separate parameter groups were assigned for empty vector and the other conditions to apply individual LFQ normalization. LFQ results were used for statistical analysis and all conditions (Flag-FBXO45, Flag-FBXO45ΔSPRY or Flag-SPRYD3) were compared with Flag-EV using an unpaired, two-tailed Student’s *t* test. In case of Flag-FBXO45 and Flag-FBXO45ΔSPRY, significantly enriched proteins were compared between those two conditions by another unpaired, two-tailed Student’s *t* test. Significance threshold was set at *p* < 0.05, with n = 4.

### Statistical analysis

All statistical analyses were performed with GraphPad Prism, version 10 (GraphPad Software, Inc.). At least three independent experiments were performed for each dataset, and each replicate was represented as individual values or as mean ± SD. Fold change data was analyzed by normalization of all values to a control group. Resulting values were log-transformed to ensure normal data distribution. Statistical significance of the logarithmically transformed values was analyzed by one-sample, two-tailed t and Wilcoxon test against the mean of the control group, which was set to 1.0. For CHX chases a two-way ANOVA was applied. While log-transformed values were used for statistical significance analysis, non-transformed values were used for visualization of fold-change data in quantification graphs. Normally distributed data, which was not normalized to a control group, was analyzed for its statistical significance by performing an unpaired, two-tailed, Student’s *t* test with Welch’s correction or ordinary one-way ANOVA for comparisons between multiple groups. All *p*-values below a threshold of 0.05 were considered statistically significant (ns > 0.05, ∗*p* < 0.05, ∗∗*p* < 0.01, ∗∗∗*p* < 0.001 and ∗∗∗∗*p* < 0.0001).

## Data availability

The MS proteomics data has been deposited in Proteomics Identification Database (PRIDE) repository with the dataset identifier PXD062852. All other data is included in this article. Further information and raw data are available upon request.

## Supporting information

This article contains supporting information.

## Conflict of Interest

The authors declare that they do not have any conflicts of interest with the content of this article.
