# Supplementary material for: The E3 ubiquitin ligase SPRYD3-MYCBP2(PAM) regulates mitotic cell fate and ubiquitination of USP11 to control spindle assembly
Source: J Biol Chem. 2025 Oct 4;301(12):110785. doi: 10.1016/j.jbc.2025.110785 (PMC12664034; doi:10.1016/j.jbc.2025.110785)
Supplement: Supporting Information Figures [file mmc1.pdf]

The E3 ubiquitin ligase SPRYD3-MYCBP2(PAM) regulates mitotic cell fate by ubiquitination of USP11 to control spindle assembly

Alexandra Rita Turi da Fonte Dias*[+] and Ingrid Hoffmann*

*Cell Cycle Control and Carcinogenesis, D345, Im Neuenheimer Feld 242, 69120 Heidelberg, Germany

[+] Faculty of Biosciences, Heidelberg University, Germany

Supporting Inforamtion PDF File includes:

- Supporting Information Figures 1-5
- Respective Supporting Information Figure Legends

Figure S-1

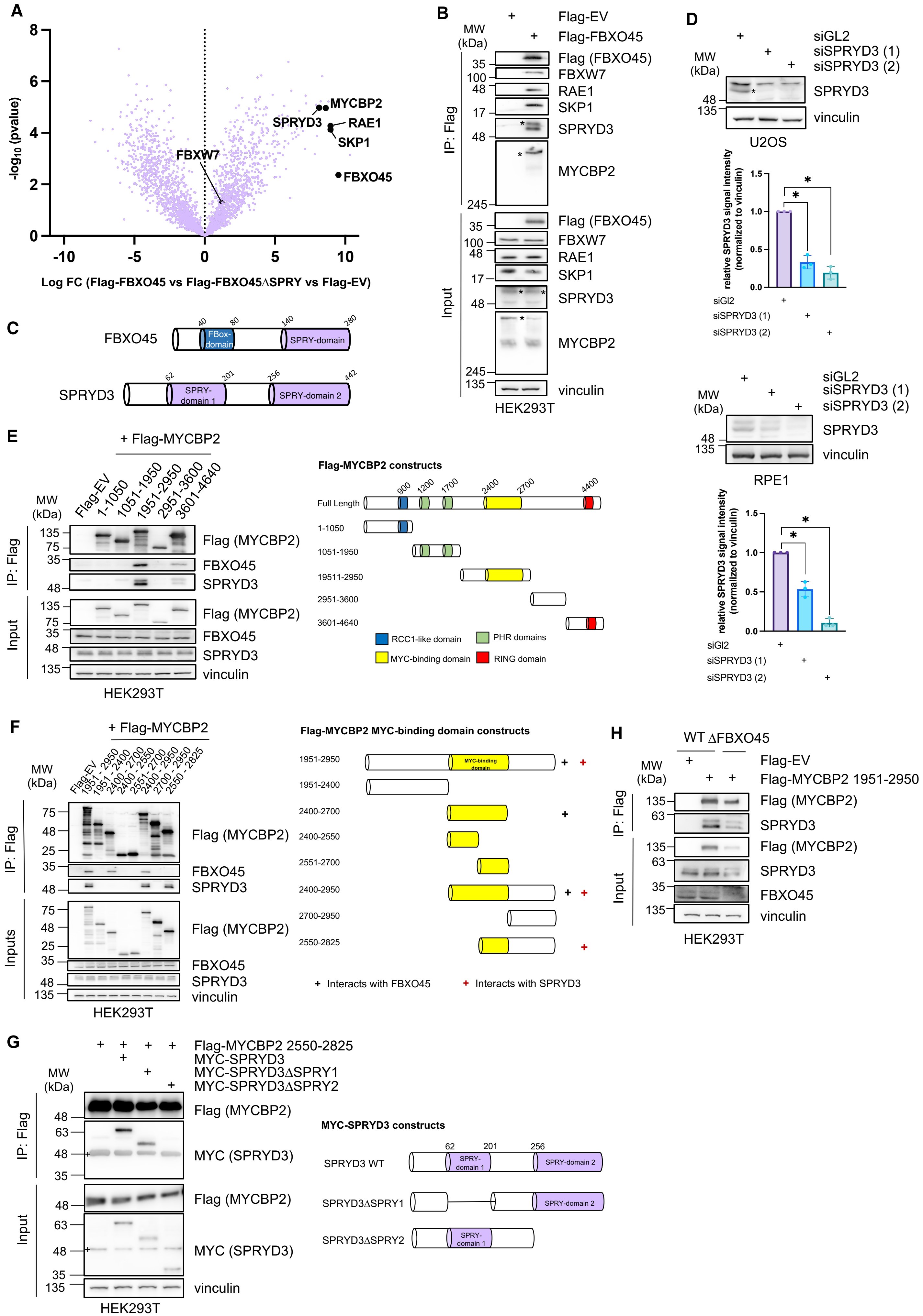

A
-log10 (pvalue)
Log FC (Flag-FBXO45 vs Flag-FBXO45ΔSPRY vs Flag-EV)
MYCBP2
SPRYD3
RAE1
SKP1
FBXW7
FBXO45
B
Flag-EV
Flag-FBXO45
MW (kDa)
IP: Flag
Input
Flag (FBXO45)
FBXW7
RAE1
SKP1
SPRYD3
MYCBP2
vinculin
HEK293T
C
FBXO45
FBox-domain
SPRY-domain
SPRYD3
SPRY-domain 1
SPRY-domain 2
D
siGL2
siSPRYD3 (1)
siSPRYD3 (2)
U2OS
RPE1
relative SPRYD3 signal intensity (normalized to vinculin)
E
+ Flag-MYCBP2
Flag-EV
1-1050
1051-1950
1951-2950
2951-3600
3601-4640
Flag (MYCBP2)
Flag-MYCBP2 constructs
Full Length
RCC1-like domain
PHR domains
MYC-binding domain
RING domain
F
Flag-MYCBP2 MYC-binding domain constructs
Interacts with FBXO45
Interacts with SPRYD3
Inputs
G
Flag-MYCBP2 2550-2825
MYC-SPRYD3
MYC-SPRYD3ΔSPRY1
MYC-SPRYD3ΔSPRY2
MYC (SPRYD3)
MYC-SPRYD3 constructs
SPRYD3 WT
SPRYD3ΔSPRY1
SPRYD3ΔSPRY2
H
WT ΔFBXO45
Flag-MYCBP2 1951-2950

Figure Legend S-1:

(A) Volcano plot demonstrating enrichment of potential FBXO45 interaction partners identified by a combined Flag-IP and MS-analysis approach. n = 4. **(B)** HEK293T WT cells were transfected with Flag-FBXO45 and incubated for 24 h. Cells were harvested for Flag-IP. Western blot analysis demonstrates co-precipitation of indicated proteins with Flag-FBXO45. * indicates SPRYD3 or MYCBP2 respectively. **(C)** Simplified schematic overview of FBXO45 and SPRYD3 and their domains. **(D)** U2OS or RPE1 cells were transfected twice with 20 nM siRNA targeting GL2 (control) or SPRYD3 24 h and 48 h after seeding. 72 h after the first transfection, cells were harvested for lysis and western blot analysis. SPRYD3 protein levels were assessed using western blot signals after vinculin-normalization and siGL2-treated control was used as 100 % reference. Relative values were log-transformed to obtain parametric distribution and applied to one sample t and Wilcoxon test, n=3. *$p < 0.05$. * indicates SPRYD3. **(E)** Flag-MYCBP2 constructs were transfected into HEK293T WT cells and overexpressed for 24 h. Cells were harvested for Flag-IP. Western blot analysis was performed to assess FBXO45 or SPRYD3 co-precipitation. **(F)** Flag-MYCBP2 MYC-binding domain truncations were ectopically expressed in HEK293T WT cells for 24 h. Follow-up procedures were executed as described in (E). **(G)** Flag-MYCBP2 2550-2825 was ectopically co-expressed with MYC-SPRYD3, MYC-SPRYD3ΔSPRY1 or MYC-SPRYD3ΔSPRY2 in HEK293T WT cells for 24 h. Cells were harvested for Flag-IP. Western blot analysis was performed to assess interaction between MYCBP2 and SPRYD3 SPRY-domains. + indicates unspecific bands. **(H)** HEK293T WT or ΔFBXO45 cells were transfected with Flag-MYCBP2 1951-2950 and incubated for 24 h. Cells were harvested for Flag-IP. Western blot analysis demonstrates SPRYD3 co-precipitation after Flag-IP in both cell lines.

Figure S-2

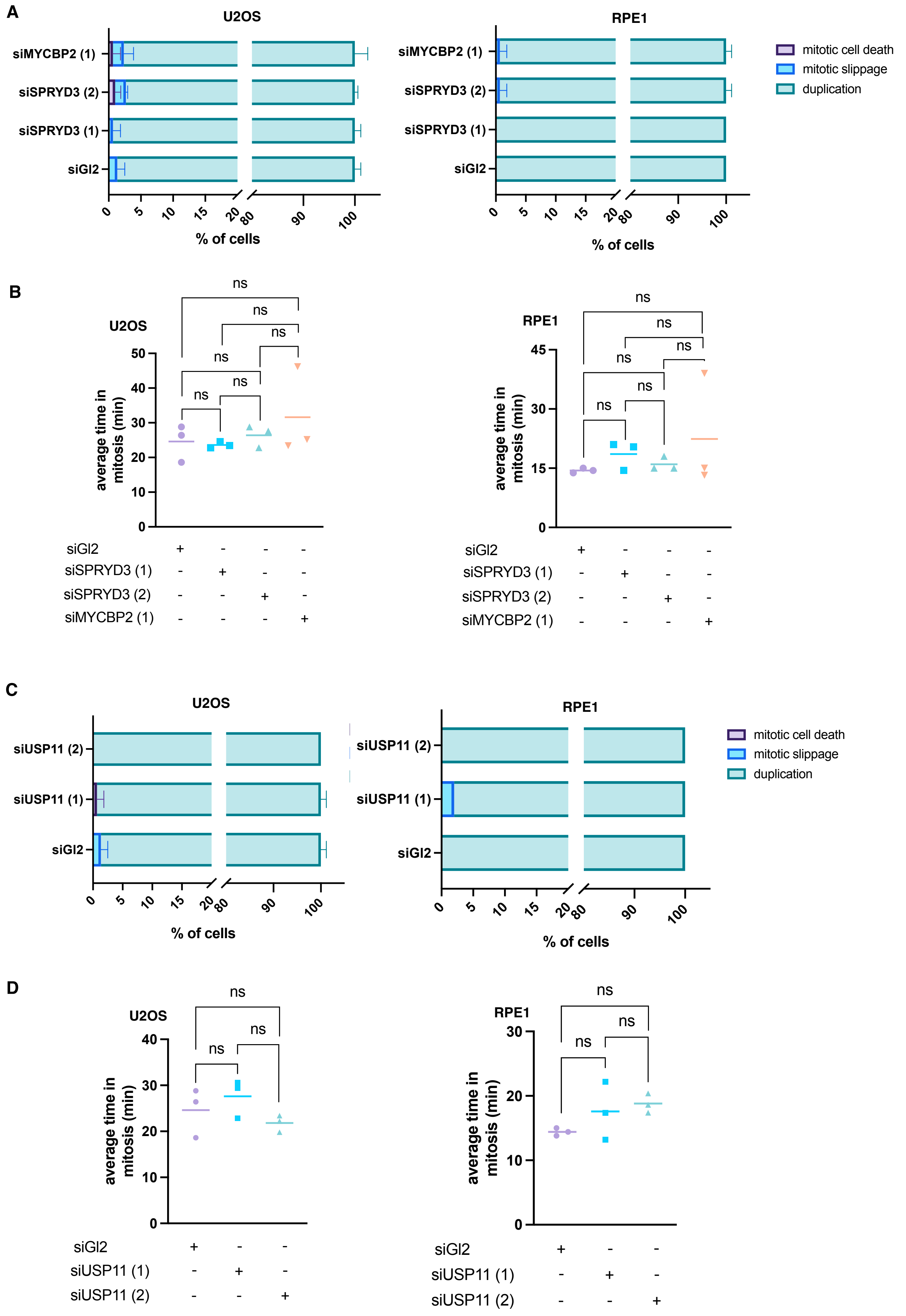

A
U2OS
siMYCBP2 (1)
siSPRYD3 (2)
siSPRYD3 (1)
siGl2
0 5 10 15 20 80 90 100
% of cells
RPE1
siMYCBP2 (1)
siSPRYD3 (2)
siSPRYD3 (1)
siGl2
0 5 10 15 20 80 90 100
% of cells
mitotic cell death
mitotic slippage
duplication
B
U2OS
ns
average time in mitosis (min)
0 10 20 30 40 50
siGl2 + - - -
siSPRYD3 (1) - + - -
siSPRYD3 (2) - - + -
siMYCBP2 (1) - - - +
RPE1
ns
average time in mitosis (min)
0 15 30 45
siGl2 + - - -
siSPRYD3 (1) - + - -
siSPRYD3 (2) - - + -
siMYCBP2 (1) - - - +
C
U2OS
siUSP11 (2)
siUSP11 (1)
siGl2
0 5 10 15 20 80 90 100
% of cells
RPE1
siUSP11 (2)
siUSP11 (1)
siGl2
0 5 10 15 20 80 90 100
% of cells
mitotic cell death
mitotic slippage
duplication
D
U2OS
ns
average time in mitosis (min)
0 10 20 30 40
siGl2 + - -
siUSP11 (1) - + -
siUSP11 (2) - - +
RPE1
ns
average time in mitosis (min)
0 10 20 30
siGl2 + - -
siUSP11 (1) - + -
siUSP11 (2) - - +

Figure Legend S-2:

(A) U2OS or RPE1 cells were transfected twice with 20 nM siRNA targeting GL2 (control), SPRYD3 or MYCBP2 24 h and 48 h after seeding. 72 h after the first transfection, live cell imaging was performed to determine mitotic cell fate of 60 cells under unperturbed conditions. *ns* > 0.05, one-way ANOVA with Dunette post hoc test, n=3. **(B)** U2OS or RPE1 cells were transfected twice with 20 nM siRNA targeting GL2 (control), SPRYD3 or MYCBP2 as described in (A). 72 h after the first transfection, live cell imaging was performed to determine time in mitosis for 60 cells. Mitotic timing was determined by visually following cells from roundup (= beginning of mitosis) until cytokinesis (=end of mitosis). *ns* > 0.05, one-way ANOVA with Dunette post hoc test, n=3 for each cell line. **(C)** U2OS or RPE1 cells were transfected twice with 20 nM siRNA targeting GL2 (control) or USP11 as described in (A). Follow-up procedures were executed as described in (A). n=3. **(D)** U2OS or RPE1 cells were transfected twice with 20 nM siRNA targeting GL2 (control) or USP11 as described in (A). Follow-up procedures were executed as described in (B).

Figure S-3

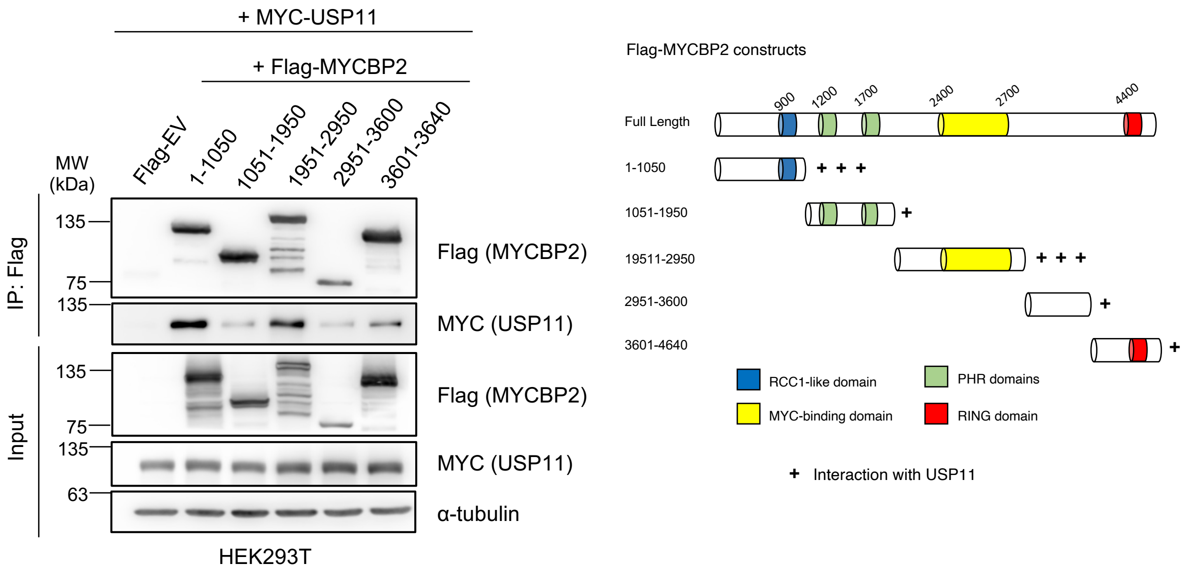

Figure Legend S-3:

Flag-MYCBP2 constructs were ectopically co-expressed with MYC-USP11 in HEK293T WT cells for 24 h. Cells were harvested for Flag-IP. Western blot analysis was performed to assess MYC-USP11 co-precipitation after Flag-IP.

Figure S-4

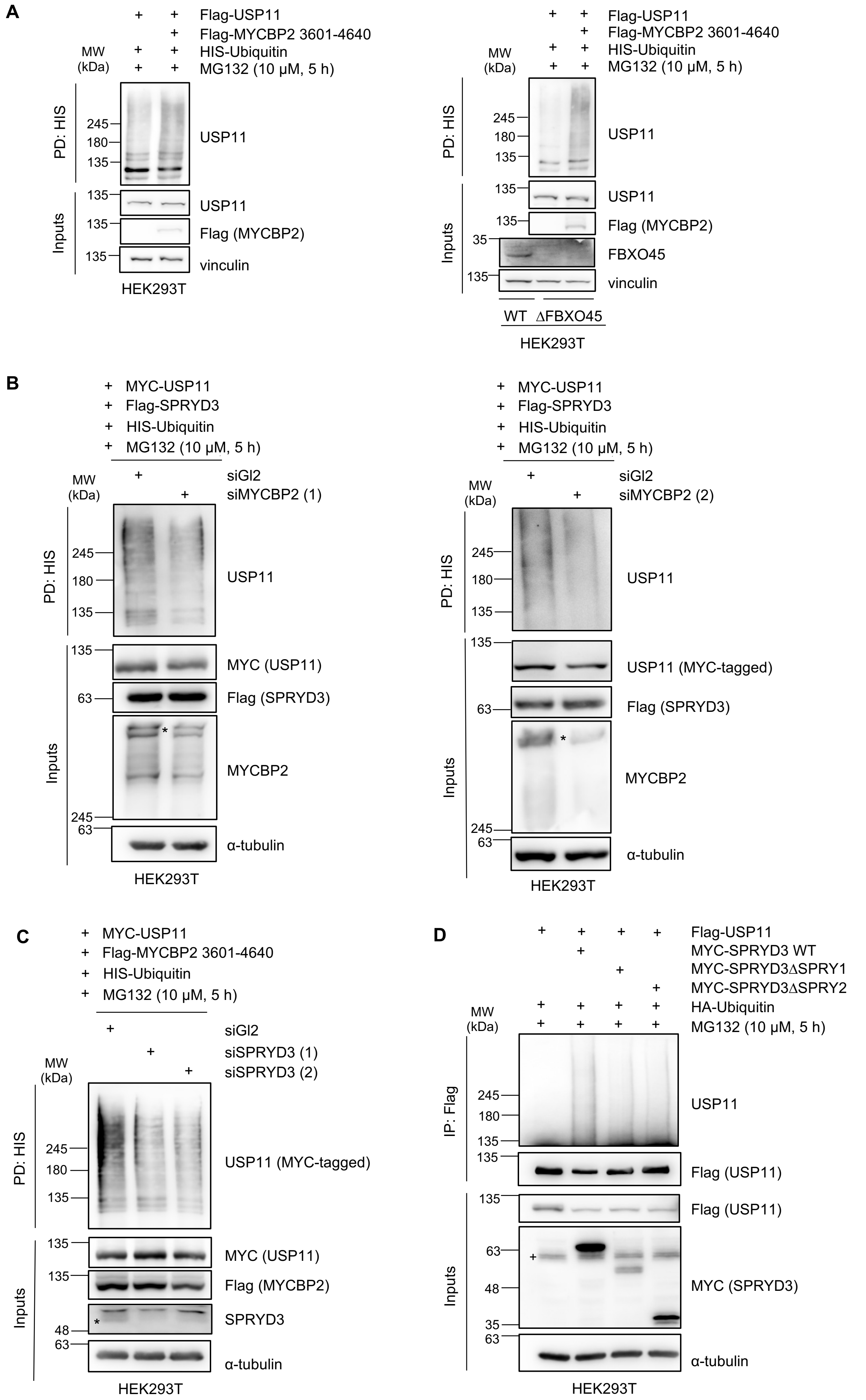

A
Flag-USP11
Flag-MYCBP2 3601-4640
HIS-Ubiquitin
MG132 (10 µM, 5 h)
MW (kDa)
PD: HIS
Inputs
245
180
135
USP11
Flag (MYCBP2)
vinculin
FBXO45
35
WT
ΔFBXO45
HEK293T
B
MYC-USP11
Flag-SPRYD3
HIS-Ubiquitin
MG132 (10 µM, 5 h)
siGl2
siMYCBP2 (1)
siMYCBP2 (2)
MYC (USP11)
USP11 (MYC-tagged)
Flag (SPRYD3)
MYCBP2
α-tubulin
63
*
C
Flag-MYCBP2 3601-4640
siSPRYD3 (1)
siSPRYD3 (2)
SPRYD3
48
D
MYC-SPRYD3 WT
MYC-SPRYD3ΔSPRY1
MYC-SPRYD3ΔSPRY2
HA-Ubiquitin
IP: Flag
Flag (USP11)
MYC (SPRYD3)

Figure Legend S-4:

(A) Flag-USP11, Flag-MYCBP2 3601-4640 and HIS-Ubiquitin were ectopically expressed in HEK293T WT or ΔFBXO45 cells for 24 h. MG132 was added 5 h prior to cell harvest for Ni-NTA pull-down. Western blot analysis was performed to assess USP11 ubiquitination status. **(B)** HEK293T WT cells were transfected twice with 20 nM siRNA targeting GL2 (= control) or MYCBP2 24 h and 48 h after seeding. 48 h after the first transfection, MYC-USP11, Flag-SPRYD3 and HIS-Ubiquitin were co-transfected and expressed for further 24 h. MG132 treatment was performed as described in (A). Cells were harvested for Ni-NTA pull-down. USP11 ubiquitination status was assessed through western blot analysis. * indicates MYCBP2. **(C)** HEK293T WT cells were transfected twice with 20 nM siRNA targeting GL2 (= control) or SPRYD3 as described in (B). 48 h after the first transfection, MYC-USP11, Flag-MYCBP2 3601-46440 and HIS-Ubiquitin were co-transfected and expressed for further 24 h. Follow-up procedures were executed as described in (B). ***** indicates SPRYD3. **(D)** Flag-USP11, MYC-SPRYD3 WT, MYC-SPRYD3ΔSPRY1 or MYC-SPRYD3ΔSPRY2 and HA-Ubiquitin were ectopically expressed in HEK293T for 24 h. MG132 treatment was performed for 5 h and cells were harvested for Flag-IP. Western blot analysis demonstrates USP11 ubiquitination upon ectopic expression of SPRYD3 WT, but not SPRYD3ΔSPRY1, which is less expressed and probably recruits less MYCBP2 (Input, lane 4) and SPRYD3ΔSPRY2, which cannot recruit MYCBP2. + indicates unspecific bands.

Figure S-5

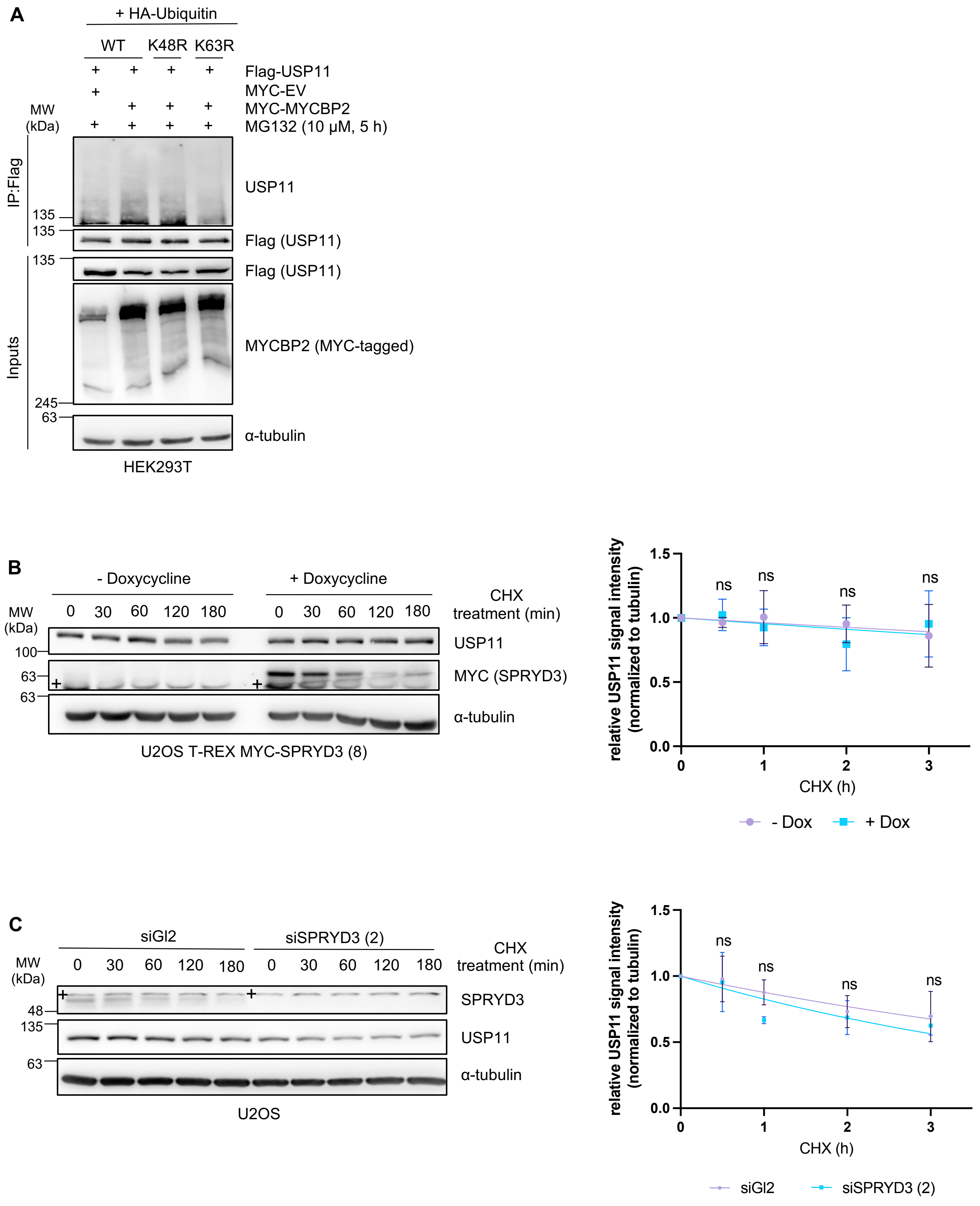
A
+ HA-Ubiquitin
WT
K48R
K63R
Flag-USP11
MYC-EV
MYC-MYCBP2
MG132 (10 µM, 5 h)
MW (kDa)
IP:Flag
Inputs
135
245
63
USP11
Flag (USP11)
MYCBP2 (MYC-tagged)
α-tubulin
HEK293T
B
- Doxycycline
+ Doxycycline
CHX treatment (min)
0 30 60 120 180
100
USP11
MYC (SPRYD3)
U2OS T-REX MYC-SPRYD3 (8)
relative USP11 signal intensity (normalized to tubulin)
CHX (h)
ns
- Dox
+ Dox
C
siGl2
siSPRYD3 (2)
48
SPRYD3
U2OS

Figure Legend S-5:

(A) Flag-USP11, MYC-MYCBP2 and HA-Ubiquitin variants were co-expressed in HEK293T WT cells for 48 h. MG132 was added 5h prior to cell harvest for Flag-IP. Western blot analysis was performed to assess Flag-USP11 ubiquitination status with different ubiquitin variants. **(B)** U2OS T-REX MYC-SPRYD3 clone (8) was treated with doxycycline for 72 h. Protein synthesis was blocked via CHX treatment (100 µg/ml) for indicated durations prior to harvest. Protein half-lives were determined by fitting to a one-phase decay model. For statistical analysis, USP11 protein levels were normalized to respective tubulin signal and values were log-transformed. Log-transformed values were applied to two-way ANOVA, n=3. *ns* > 0.05. + indicates unspecific bands. **(C)** U2OS cells were transfected twice with siRNA targeting GL2 (control) or SPRYD3 24 h and 48 h after seeding. 72 h after the first transfection, protein synthesis was blocked via CHX treatment (100 µg/ml) for indicated durations prior to harvest. Protein half-lives were determined by fitting to a one-phase decay model. Statistical analysis was carried out as described in (B). n=3. ns > 0.05. + indicates unspecific bands.
